# Association of Wearable Activity Monitors With Assessment of Daily Ambulation and Length of Stay Among Patients Undergoing Major Surgery

**DOI:** 10.1001/jamanetworkopen.2018.7673

**Published:** 2019-02-01

**Authors:** Timothy J. Daskivich, Justin Houman, Mayra Lopez, Michael Luu, Philip Fleshner, Karen Zaghiyan, Scott Cunneen, Miguel Burch, Christine Walsh, Guy Paiement, Thomas Kremen, Harmik Soukiasian, Andrew Spitzer, Titus Jackson, Hyung L. Kim, Andrew Li, Brennan Spiegel

**Affiliations:** 1 Division of Urology, Department of Surgery, Cedars-Sinai Medical Center, Los Angeles, California; 2 Cedars-Sinai Center for Outcomes Research and Education (CS-CORE), Cedars-Sinai Medical Center, Los Angeles, California; 3 Division of Colorectal Surgery, Department of Surgery, Cedars-Sinai Medical Center, Los Angeles, California; 4 Division of Minimally Invasive Surgery, Department of Surgery, Cedars-Sinai Medical Center, Los Angeles, California; 5 Department of Obstetrics and Gynecology, Cedars-Sinai Medical Center, Los Angeles, California; 6 Department of Orthopedic Surgery, Cedars-Sinai Medical Center, Los Angeles, California; 7 Division of Thoracic Surgery, Department of Surgery, Cedars-Sinai Medical Center, Los Angeles, California; 8 Division of Health Services Research, Department of Medicine, Cedars-Sinai Health System, Los Angeles, California; 9 Department of Health Policy and Management, UCLA Fielding School of Public Health, Los Angeles

## Abstract

**Importance:**

Early postoperative ambulation is vital to minimizing length of stay (LOS), but few hospitals objectively measure ambulation to predict outcomes. Wearable activity monitors have the potential to transform assessment of postoperative ambulation, but key implementation data, including whether digitally monitored step count can identify patients at risk for poor efficiency outcomes, are lacking.

**Objectives:**

To define the distribution of digitally measured daily step counts after major inpatient surgical procedures, to assess the accuracy of physician assessment and ordering of ambulation, and to quantify the association of digitally measured step count with LOS.

**Design, Setting, and Participants:**

Prospective cohort study at Cedars-Sinai Medical Center, an urban tertiary referral center. Participants were patients undergoing 8 inpatient operations (lung lobectomy, gastric bypass, hip replacement, robotic cystectomy, open colectomy, abdominal hysterectomy, sleeve gastrectomy, and laparoscopic colectomy) from July 11, 2016, to August 30, 2017.

**Interventions:**

Use of activity monitors to measure daily postoperative step count.

**Main Outcomes and Measures:**

Operation-specific daily step count, daily step count by physician orders and assessment, and a prolonged LOS (>70th percentile for each operation).

**Results:**

Among 100 patients (53% female), the mean (SD) age was 53 (18) years, and the median LOS was 4 days (interquartile range, 3-6 days). There was a statistically significant increase in daily step count with successive postoperative days in aggregate (*r* = 0.55; 95% bootstrapped CI, 0.47-0.62; *P* < .001) and across individual operations. Ninety-five percent (356 of 373) of daily ambulation orders were “ambulate with assistance,” although daily step counts ranged from 0 to 7698 steps (0-5.5 km) under this order. Physician estimation of ambulation was predictive of the median step count (*r* = 0.66; 95% bootstrapped CI, 0.59-0.72; *P* < .001), although there was substantial variation within each assessment category. For example, daily step counts ranged from 0 to 1803 steps (0-1.3 km) in the “out of bed to chair” category. Higher step count on postoperative day 1 was associated with lower odds of prolonged LOS from 0 to 1000 steps (odds ratio [OR], 0.63; 95% CI, 0.45-0.84; *P* = .003), with no further decrease in odds after 1000 steps (OR, 0.99; 95% CI, 0.75-1.30; *P* = .80).

**Conclusions and Relevance:**

In this study, digitally measured step count up to 1000 steps on postoperative day 1 was associated with lower probability of a prolonged LOS. Wearable activity monitors improved the accuracy of assessment of daily step count over the current standard of care, providing an opportunity to identify patients at risk for poor efficiency outcomes.

## Key Points

**Question:**

Do wearable activity monitors measuring step count after major surgery improve assessment of daily ambulation and predict length of stay?

**Findings:**

In this cohort study of 100 patients undergoing major surgery at Cedars-Sinai Medical Center, activity monitors were associated with improved accuracy of assessment of step count. Higher step count up to 1000 steps on postoperative day 1 was associated with significantly lower odds of prolonged length of stay, with no further decrease in odds after 1000 steps.

**Meaning:**

In this study, activity monitors improved assessment of daily ambulation, and daily step count was associated with length of stay, providing an opportunity to identify patients at risk for poor efficiency outcomes.

## Teaser

This cohort study among patients undergoing major surgery defines the distribution of digitally measured daily step counts after major inpatient surgical procedures, assesses the accuracy of physician assessment and ordering of ambulation, and quantifies the association of digitally measured step count with postoperative length of stay in the hospital.

## Introduction

Ambulatory status is a fundamental factor in management of the postoperative surgical inpatient and has been linked to both key clinical (eg, deep venous thromboembolism)^1,2,3^ and efficiency (eg, cost of inpatient care and length of stay [LOS])^4,5,6,7,8^ outcomes. Yet, despite its central role in recovery and disposition, daily assessment of ambulation is imprecise and is an often neglected component of nursing care, missed 76% to 89% of the time.^9,10,11,12^ Most often, assessments of daily ambulation rely on patient report, which may be inaccurate, or nursing accounts, which are difficult to ascertain due to frequent handoffs in care. Given the high stakes for poor ambulation, surgical teams have a need for better information regarding postoperative ambulation, including a simple method for quantification of an objective ambulation goal linked to a relevant clinical outcome.

The advent of commercially available, wearable activity monitors allows quantitative digital monitoring of daily postoperative ambulation in a manner that is both feasible and scalable. Yet, while quantifying ambulation with activity monitors may be achievable, there is a lack of key data to permit implementation in the clinical setting. First, there is no information on the distribution of daily step count after individual surgical procedures, limiting the ability of patients and surgeons to understand what is average, above average, and below average for a given operation. Second, there are no data analyzing the current standard of care for assessment and ordering of ambulation to determine its accuracy for monitoring and execution of ambulation orders. Third, there is no evidence linking quantity of digitally monitored steps to relevant outcomes, which would provide a rationale for more granular assessment of ambulation over the current standard of care.

To address this issue, we conducted a prospective cohort study using a commercially available, wearable activity monitor to digitally measure step count after 8 major surgical procedures. Our study had the following 3 main objectives: (1) to define the distribution of digitally measured daily step count across surgical procedures, (2) to assess the correlation of physician daily orders for ambulation and physician estimates of daily ambulation with digitally measured step count, and (3) to quantify the association of digitally measured step count with probability of a prolonged LOS. We hypothesized that physician orders for ambulation and estimates of ambulation would be poorly correlated with digitally monitored step count and that lower step counts would be associated with higher probability of a prolonged LOS. This information would provide a rationale for using wearable activity monitors in the clinical setting and provide foundational data for future interventional studies.

## Methods

### Participants

We recruited 128 patients undergoing 8 major inpatient operations (lung lobectomy, gastric bypass, hip replacement, robotic cystectomy, open colectomy, abdominal hysterectomy, sleeve gastrectomy, and laparoscopic colectomy) from July 11, 2016, to August 30, 2017, at Cedars-Sinai Medical Center, an urban tertiary referral center. Participants were required to be 18 years or older. Individuals were excluded if they were admitted to the intensive care unit after surgery; used a walker, cane, or wheelchair at baseline; were unable to walk due to physical limitation; or were unable to maintain the activity monitor in place. Participants were recruited during outpatient surgical consultation visits, and written informed consent was obtained by participating coinvestigators (T.J.D., J.H., P.F., K.Z., S.C., M.B., C.W., G.P., T.K., H.S., A.S., T.J., H.L.K., and A.L.) or study staff (M. Lopez). Among 128 individuals who consented, 8 were excluded owing to cancellation of surgery, 3 owing to refusal to participate after surgery, and 15 owing to loss of the activity monitor or data. An additional 2 patients were dropped from the analysis because they were extreme outliers (LOS >30 days). The final analytic sample was 100 patients. This study followed the Strengthening the Reporting of Observational Studies in Epidemiology (STROBE) reporting guideline. Study approval was granted by the Cedars-Sinai Medical Center Institutional Review Board. The study was registered at ClinicalTrials.gov (NCT02741895).

### Study Procedures and Device Management

After the patient was awake and alert after surgery, a study team member placed a Fitbit Charge (Fitbit Inc) activity monitor on his or her wrist. Participants were encouraged to keep the device in place throughout their hospitalization except while showering. Daily step count was passively monitored for the duration of hospitalization. Activity monitors were recharged as needed (generally every 5-7 days). Data from the activity monitor were uploaded via a secure Bluetooth connection at the time of discharge.

### Variables

#### Activity Monitor Data

Daily step count was measured as number of steps taken over 24 hours starting at 6 am on the indicated postoperative day. Postoperative day 0 was defined as the time from placement of the device until 6 am on the first day after surgery. Step count to kilometer conversions were based on a weighted average of gait length based on the sex distribution of our sample (men, 2.5 feet/step; women, 2.2 feet/step).

#### Surgeon Estimate of Step Count

Surgeons were asked to record their estimates of the patient’s ambulation the day prior in progress notes documenting morning rounds. Surgeons were asked to use standardized terms to describe ambulation based on the following commonly used terminology: no ambulation; out of bed to chair; out of bed to ambulated once daily, twice daily, or 3 times daily; or ambulated ad libitum. We intentionally opted not to ask clinicians to give a numeric estimate of step count given the lack of a well-recognized reference.

#### Ordered Ambulation Regimen

Ordered ambulation regimen for each postoperative day as entered by the clinician team was retrospectively ascertained from the medical record. Options in the electronic formulary were (1) bed rest, (2) out of bed to chair, (3) ambulate with assistance, and (4) ambulate ad libitum.

#### Additional Data

We collected sociodemographic (age, sex, and race/ethnicity) and clinical (body mass index and comorbid conditions) data via review of the medical record.

### Statistical Analysis

Baseline patient characteristics were compared across operation type using 1-way analysis of variance for continuous variables. Pearson χ^2^ test was used for categorical variables.

To illustrate the distribution of wearable, digitally monitored step count by postoperative day, we created box-and-whiskers plots in aggregate across all operations and individual operations. Pearson product moment correlation analysis was used to identify correlation between daily step count and postoperative days. We used bootstrapping with 1000 replicates to calculate bias-corrected and accelerated 95% CIs. Bartlett test of homogeneity of variances was used to test for differences in variances among daily step counts and postoperative days.^13^

To visualize the association of digitally measured step count with ordered ambulation regimen and with surgeons’ assessment of daily ambulation, we created box-and-whiskers plots showing digitally monitored step count across categories of ordered ambulation and categories of daily ambulation estimates. To determine if digitally monitored daily step count is associated with operation-specific LOS, we used a multivariable binomial logistic regression model.^14^ Because this outcome is operation specific, we constructed operation-specific dichotomization of LOS at 70th, 80th, and 90th percentiles for each operation type, in which LOS above the selected percentile is considered a prolonged LOS. We used step count on postoperative day 1 as our primary predictor, adjusting for operation type, age, sex, race/ethnicity, and Charlson-Deyo comorbidity score. Body mass index was not included as a covariate because it was collinear with operation type. Because steps taken violated the assumption of linearity, this variable was allowed flexibility and was modeled as a restricted cubic spline with 3 knots corresponding to the 10th, 50th, and 90th quantiles as recommended by Harrel.^15^ Variable selection was performed using a stepwise method optimizing for the Akaike information criterion. Collinearity was checked by the variance inflation factor. Change point in daily step count and its association with long LOS was estimated using a piecewise linear regression model.^16^ Probability estimates by day 1 step count were constructed in aggregate and then stratified by specific operation. We also created identical models using step counts from postoperative day 2. We did not analyze data beyond postoperative day 2 due to low sample size.

All statistical analyses were performed with R statistical software (version 3.4; R Foundation for Statistical Computing) using 2-sided tests. *P* < .05 was considered statistically significant. The coding script is included in the eAppendix in the Supplement.

## Results

Our final analytic sample included 100 patients. The mean (SD) age was 53 (18) years. Most participants were female (53%) and of white race/ethnicity (55%) and had a Charlson-Deyo comorbidity score of 0 (62%) (Table). The median LOS was 4 days (interquartile range [IQR], 3-6 days). In general, participants undergoing lung lobectomy, hip replacement, and robotic cystectomy were older; those undergoing gastric bypass and sleeve gastrectomy had higher body mass index; and those undergoing robotic cystectomy had longer LOS compared with other operations.

**Table.** Sample Characteristics by Operation Type^a^

Variable	Total (N = 100)	Lung Lobectomy (n = 6)	Gastric Bypass (n = 6)	Hip Replacement (n = 15)	Robotic Cystectomy (n = 12)	Open Colectomy (n = 16)	Abdominal Hysterectomy (n = 11)	Sleeve Gastrectomy (n = 19)	Laparoscopic Colectomy (n = 15)
Age, mean (SD), y	53 (18)	70 (8)	37 (10)	62 (13)	70 (10)	43 (18)	56 (11)	44 (15)	47 (20)
Sex, No./total No. (%)									
Male	47/100 (47)	2 (33)	0	7 (47)	9 (75)	13 (81)	0	5 (26)	11 (73)
Female	53/100 (53)	4 (67)	6 (100)	8 (53)	3 (25)	3 (19)	11 (100)	14 (74)	4 (27)
Race/ethnicity, No./total No. (%)									
White	55/100 (55)	1 (17)	1 (17)	10 (67)	8 (67)	12 (75)	5 (45)	7 (37)	11 (73)
African American	12/100 (12)	0	3 (50)	2 (13)	1 (8)	0	3 (27)	3 (16)	0
Hispanic	20/100 (20)	2 (33)	2 (33)	1 (7)	0	4 (25)	3 (27)	7 (37)	1 (7)
Asian American	5/100 (5)	3 (50)	0	1 (7)	0	0	0	0	1 (7)
Middle Eastern	8/100 (8)	0	0	1 (7)	3 (25)	0	0	2 (11)	2 (13)
BMI, mean (SD)	31 (12)	28 (4)	49 (11)	30 (5)	27 (3)	23 (3)	29 (7)	46 (12)	23 (4)
Charlson-Deyo comorbidity score, No./total No. (%)									
0	62/100 (62)	1 (17)	5 (83)	11 (73)	1 (8)	13 (81)	6 (55)	11 (58)	14 (93)
1	12/100 (12)	2 (33)	1 (17)	1 (7)	0	0	0	8 (42)	0
≥2	26/100 (26)	3 (50)	0	3 (20)	11 (92)	3 (19)	5 (45)	0	1 (7)
Length of stay, median (IQR), d	4 (3-6)	3 (2-4)	4 (3-4)	4 (3-4)	9 (6-9)	5 (4-8)	4 (4-5)	3 (2-3)	4 (4-6)

Abbreviations: BMI, body mass index (calculated as weight in kilograms divided by height in meters squared); IQR, interquartile range.

^a^ *P* < .001 for all comparisons. *P* values were calculated by 1-way analysis of variance for continuous variables and by χ^2^ test for categorical variables.

**Figure 1.**
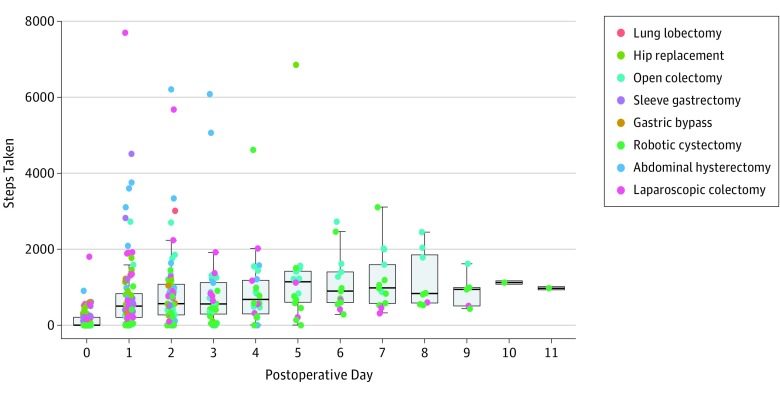
Digitally Monitored Step Count by Postoperative Day Across All Surgical Procedures Boxes show the first quartile (lower border of the box), median (thick horizontal line within the box), and third quartile (top border of the box). Whiskers extending from the top and bottom borders of the box show the range of nonoutlier values. The individual points outside the whisker boundaries indicate outlier values that are less than the first quartile or greater than the third quartile by 1.5 times the interquartile range.

Box-and-whiskers plots showing digitally monitored daily step count by postoperative day across all operations demonstrated a gradual increase in step count with each successive postoperative day until a plateau at approximately postoperative day 5 (Figure 1). The median daily step count increased from 0 (IQR, 0-211) on day 0, to 497 (IQR, 815-830) on day 1, to 565 (IQR, 956-1078) on day 2, to 566 (IQR, 921-1118) on day 3, to 676 (IQR, 902-1182) on day 4, and to 1136 (IQR, 1257-1418) on day 5. Spearman rank correlation analysis showed a statistically significant increase in step count with successive postoperative days (*r* = 0.55; 95% bootstrapped CI, 0.47-0.62; *P* < .001). There was variability in the range of steps taken, especially among the early postoperative days. For example, digitally monitored steps on postoperative day 1 ranged from 0 to 7698 steps (0-5.5 km). Bartlett test of homogeneity of variances showed significant differences in variances of steps taken among all postoperative days, with more variance observed at the early postoperative days (k = 187.21, *P* < .001).

**Figure 2.**
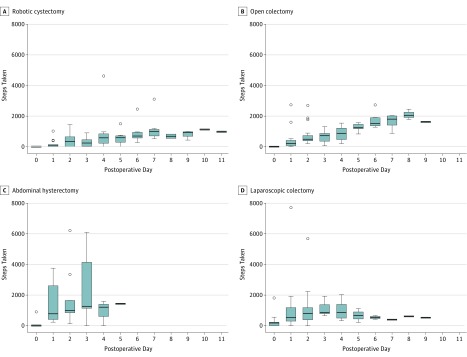
Digitally Monitored Step Count by Postoperative Day and Operation Type Boxes show the first quartile (lower border of the box), median (thick horizontal line within the box), and third quartile (top border of the box). Whiskers extending from the top and bottom borders of the box show the range of nonoutlier values. The individual points outside the whisker boundaries indicate outlier values that are less than the first quartile or greater than the third quartile by 1.5 times the interquartile range.

Box-and-whiskers plots showing digitally monitored daily step count by postoperative day across operations revealed differences in step count according to operation type (Figure 2). In general, Spearman rank correlation showed significant increases in step count with successive postoperative days, echoing the trend seen in the aggregate analysis. Operation-specific Bartlett tests of homogeneity of variances showed significant variance in step count among all operations except lung lobectomy and gastric bypass, with higher variance during the early postoperative days (Figure 2).

**Figure 3.**
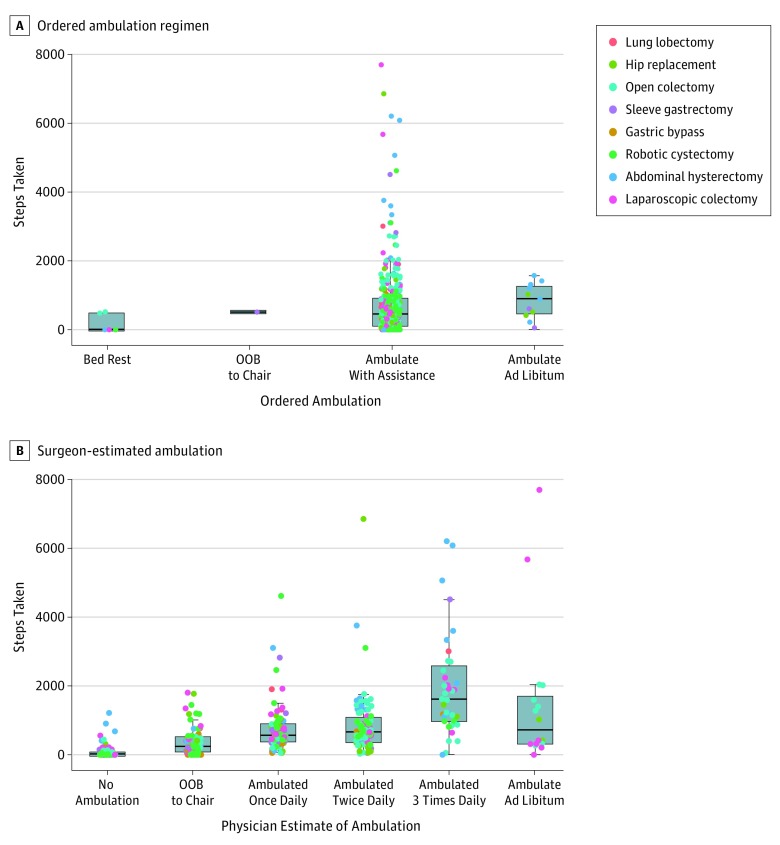
Digitally Monitored Step Count by Ordered Ambulation Regimen and Surgeon-Estimated Ambulation Boxes show the first quartile (lower border of the box), median (thick horizontal line within the box), and third quartile (top border of the box). Whiskers extending from the top and bottom borders of the box show the range of nonoutlier values. The individual points outside the whisker boundaries indicate outlier values that are less than the first quartile or greater than the third quartile by 1.5 times the interquartile range. OOB indicates out of bed.

Box-and-whiskers plots depicting digitally monitored daily step count by ordered daily ambulation regimen demonstrated uniformity in ordered ambulation regimen despite a wide range of steps taken (Figure 3A). For example, 95% (356 of 373) of daily ambulation orders were ambulate with assistance, which included a range of step counts from 0 to 7698 (0-5.5 km).

Surgeon estimates indicating higher levels of daily ambulation were correlated with higher median digitally monitored step count (Figure 3B) (*r* = 0.66; 95% bootstrapped CI, 0.59-0.72; *P* < .001). However, there was a wide range of step counts within these categories. For example, patients categorized by their surgeons as being out of bed to chair had step counts ranging from 0 to 1803 steps (0-1.3 km).

**Figure 4.**
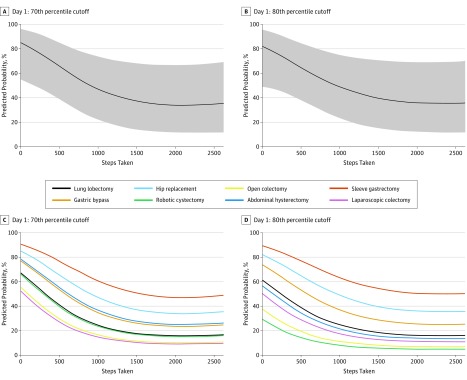
Predicted Probabilities of Prolonged Length of Stay by Postoperative Day 1 Step Count A, Operation-specific length of stay longer than the 70th percentile (data include all operations). B, Operation-specific length of stay longer than the 80th percentile (data include all operations). C, Operation-specific length of stay longer than the 70th percentile (by operation). D, Operation-specific length of stay longer than the 80th percentile (by operation). Shaded areas represent 95% CIs.

Multivariable logistic regression models showed that higher digitally monitored step count on postoperative day 1 was associated with lower probability of a prolonged LOS from 0 to 1000 steps (Figure 4). For every 100 steps taken, there was a 3.7% reduction in probability of an operation-specific prolonged LOS (LOS >70th percentile) (odds ratio [OR], 0.63; 95% CI, 0.45-0.84; *P* = .003). No significant reduction in probability of a prolonged LOS was observed past 1000 steps (OR, 0.99; 95% CI, 0.75-1.30; *P* = .80). Identical models for postoperative day 2 also showed similar associations with LOS but were not statistically significant (eFigure 1 in the Supplement).

## Discussion

This study provides implementation data that support using wearable activity monitors to track postoperative ambulation after major surgery. Our data reveal an association across operations between digitally monitored step count in the early postoperative period and LOS. We found that higher number of steps on postoperative day 1 was linearly associated with a decreased probability of a prolonged LOS until a step count of 1000 steps, beyond which there was no further decrease. For each successive increase in 100 steps toward the goal of 1000 steps, there was a 3.7% reduction in probability of the LOS being in the upper 70th percentile for a given operation. This association held up despite correction for operation type in the multivariable model, and the magnitudes of association were similar across different operations (Figure 4C and D), suggesting a generalizable finding independent of operation type. Furthermore, the association was similar for postoperative day 2, although it was a nonsignificant association. These data suggest a 1000-step daily goal for ambulation in the early postoperative period after major surgery, similar to the 10 000-step daily goal that has been espoused for healthy individuals using activity monitors.^17^ While we cannot assume that the association between digitally monitored postoperative step count and LOS is causal, our findings provide the critical foundation for future interventional studies by identifying a clinically meaningful (if ambitious) daily step count goal.

Our study also shows that activity monitors improve the accuracy of assessment of ambulation over the current standard of care. While physician estimates of daily ambulation applying categories typically used in clinical practice were in general associated with average step count, there was wide variability in number of steps taken for a given estimate. For example, among patients who were estimated to be out of bed to chair, the range of digitally monitored step count was 0 to 1803 steps (0-1.3 km). That physicians are not more accurately measuring such a critical determinant of key outcomes seems outdated in the era of digital medicine, although this assertion is contingent on the finding that such granularity of assessment is clinically meaningful. Because small differences in daily step count up to 1000 steps are associated with substantial differences in LOS, this degree of inaccuracy may indeed be clinically relevant.

Another key finding is that there is little variability in orders for daily ambulation by clinician teams, which is illustrative of the lack of precision with which ambulation is now managed. Our data showed that 95% (356 of 373) of orders were to ambulate with assistance despite other available orders for bed rest, out of bed to chair, and ambulate ad libitum. Among those ordered to ambulate with assistance, actual step count ranged from 0 to 7698 steps (0-5.5 km). It is possible that the lack of granularity in ambulation orders may be partially contributing to the wide variation in steps taken under a given order. Yet, it is not surprising that ordering of ambulation is vague given the heretofore absence of technology to precisely monitor step count and lack of specific daily step count goals. If a specified step count is important to outcomes, as our data suggest, both the ordering and monitoring of ambulation will need to be modified to target step count goals that are associated with clinically meaningful outcomes (ie, 1000 steps).

The concept of using activity monitors to measure postoperative ambulation is scalable and can be digitally integrated into the electronic medical record (EMR) to allow for real-time feedback to patients and surgical teams. Although pedometers could be used to monitor step count, the process of integrating serial pedometer measurement into clinical workflows would be prohibitively cumbersome. In contrast, digital interfaces between activity monitors, the EMR, and software packages to provide visualizations for feedback have the potential to make this process feasible. A successful interface has previously been demonstrated between activity monitors and the Epic (Epic Systems) EMR used at Cedars-Sinai Medical Center, allowing step count data from the device to passively populate a data field within the EMR.^18^ Two of us (T.J.D. and B.S.) have also developed a feedback system in which an activity monitor measures step count in real time, stores data on a database compliant with the Health Insurance Portability and Accountability Act of 1996, and then translates information to a user-friendly visualization accessible through in-room television monitors (eFigure 2 in the Supplement). Such systems will be crucial to integrating step count into busy workflows and providing a platform for feedback to patients, nurses, and physicians about progress toward step count goals.

Because most patients and clinicians have no reference for translating step counts into physical distance, we also believe that the transition to integrating granular step count into practice will require benchmarking of typical postoperative ambulation routes into step count distances. To this end, we have mapped out the floors of our surgical units to allow physicians and patients to plan ambulation regimens to reach daily goals. For example, a lap around the nursing unit at Cedars-Sinai Medical Center takes approximately 250 steps; hence, 4 laps around the unit throughout the day will allow the patient to reach the daily goal of 1000 steps. Activity-based courses benchmarked for step counts may also further engage patients to reach daily goals. For example, we have leveraged the unique museum-quality art collection at Cedars-Sinai Medical Center to create step count–benchmarked art tours, providing curated narrations about art pieces around the surgical units through a downloadable smartphone application. Activity-based routes could alternatively incorporate other media, such as biographies, moments in sports, or local history. Regardless, tying step counts to physical landmarks in the hospital will help patients and physicians better conceptualize step count and apply it to achievement of daily goals.

There are some practical considerations that should also be considered when implementing this technology. First, patients should be cautioned to wear activity monitors on the opposite hand if they are carrying an intravenous pole while walking to appropriately capture steps. Second, activity monitors require periodic charging (every 5 days for the activity monitor used herein), which will require staffing workflows to ensure continuous charge. Third, during our study, 4 devices were lost due to participants inadvertently taking them home. Given the availability and ubiquity of these devices, patients are more likely to remove them from the hospital than other durable medical equipment. Fourth, during our study, 3 patients encountered data loss due to device or user malfunction.

### Limitations

Our study has some limitations. First, our study design did not allow for assessment of causality between digitally monitored step count and LOS. Because numerous factors (eg, pain and presence of ileus) influence LOS, it is likely that increasing step count alone will not result in improvement. However, the fact that this simple variable has an association with LOS after controlling for relevant covariates underscores its ability to at least identify patients at risk for poor outcomes and target them for interventions. To this end, we are conducting a randomized clinical trial to evaluate whether our feedback system (based on a 1000-step daily goal and art tour approach) improves step count and decreases LOS and readmissions. Second, given the limited sample size in our study, we were underpowered to include all variables in our multivariable model. However, we found a significant association between step count and LOS across sensitivity analyses that included all covariates, a limited subset of covariates based on model selection, and a parsimonious model that included only operation type. Third, different activity monitors may have different thresholds for step detection and hence be associated with different daily ambulation goals. Fourth, although we compared physician assessment of daily step count with activity monitor–measured step count, we did not conduct a parallel analysis for nursing assessments of step count. Fifth, we did not account for difference in gait length or ambulation patterns in our measurements of step count, which may theoretically influence generalizability of our step count thresholds. Sixth, we did not censor daily step counts for the time of discharge, so patients who were discharged early in the day may have bias toward lower step counts on the day of discharge; however, the direction of this bias would oppose the observed association between step count and LOS, making our hypothesis harder (not easier) to prove.

## Conclusions

This study provides key implementation data and a rationale for integrating activity monitors into monitoring of postoperative ambulation after major surgery. To our knowledge, our study is the first to date to quantify the association of digitally monitored postoperative daily step count and LOS after major surgery, showing a linear decrease in probability of a prolonged LOS up to 1000 steps, after which there is no further decrease. These data suggest a 1000-step daily goal for postoperative ambulation after major surgery. We also show that the current standard of care for assessment and ordering of ambulation is inaccurate to a degree that may influence clinical outcomes. Activity monitors provide an inexpensive platform for more precise assessment, ordering, and monitoring of step count toward evidence-based daily goals and may indeed become a sixth vital sign for surgical teams.

## Notes

Supplement. **eFigure 1.** Predicted Probabilities of Prolonged Length of Stay by Postoperative Day 2 Step Count **eFigure 2.** Feedback Visualization **eAppendix.** R Script
