# Supplementary material for: Association of Wearable Activity Monitors With Assessment of Daily Ambulation and Length of Stay Among Patients Undergoing Major Surgery
Source: JAMA Netw Open. 2019 Feb 1;2(2):e187673. doi: 10.1001/jamanetworkopen.2018.7673 (PMC6484591; doi:10.1001/jamanetworkopen.2018.7673)
Supplement: Supplement. — eFigure 1. Predicted Probabilities of Prolonged Length of Stay by Postoperative Day 2 Step Count eFigure 2. Feedback Visualization eAppendix. R Script [file jamanetwopen-2-e187673-s001.pdf]

# Supplementary Online Content

Daskivich TJ, Houman J, Lopez M, et al. Association of wearable activity monitors with assessment of daily ambulation and length of stay among patients undergoing major surgery. *JAMA Netw Open*. 2019;2(2):e187673. doi:10.1001/jamanetworkopen.2018.7673

**eFigure 1.** Predicted Probabilities of Prolonged Length of Stay by Postoperative Day 2 Step Count

**eFigure 2.** Feedback Visualization

**eAppendix.** R Script

This supplementary material has been provided by the authors to give readers additional information about their work.

© 2019 Daskivich TJ et al. *JAMA Network Open*.

**eFigure 1.** Predicted Probabilities of Prolonged Length of Stay by Postoperative Day 2 Step Count

Legend: (A) Operation-specific LOS longer than 70th percentile (data includes all operations); (B) Operation-specific LOS longer than 80th percentile (data includes all operations); (C) Operation-specific LOS longer than 70th percentile (by operation); (D) Operation-specific LOS longer than 80th percentile (by operation). Grey area indicates 95% confidence intervals.

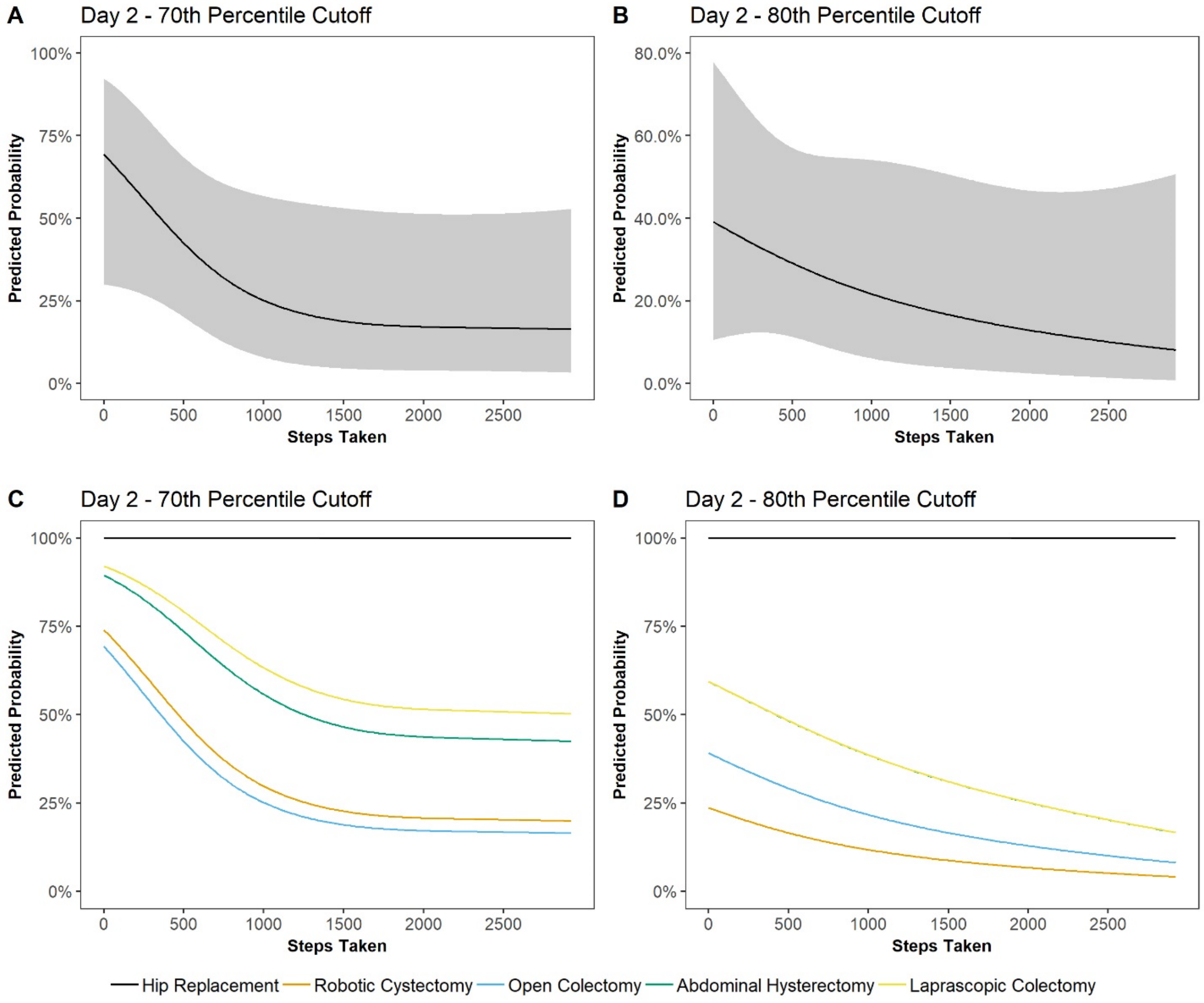


© 2019 Daskivich TJ et al. *JAMA Network Open*.

**eFigure 2.** Feedback Visualization

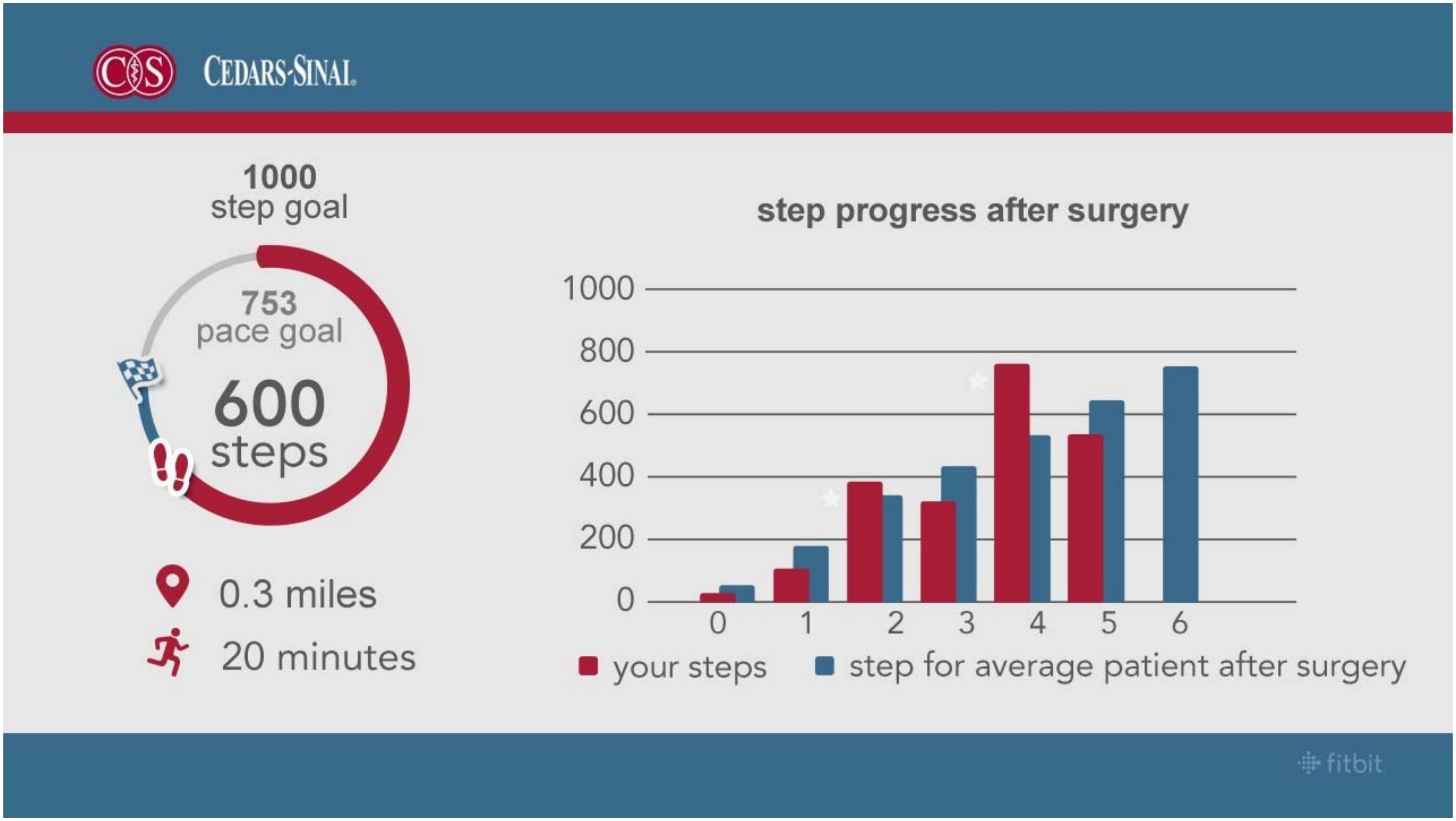


Reproduced with permission.

© 2019 Daskivich TJ et al. *JAMA Network Open*.

**eAppendix.** R Script

```
library(tidyverse)

## read in the data
df <- readxl::read_excel('data/Fitbit Study Spreadsheet 11.1.17.xlsx') %>%
janitor::clean_names()

## remove empty rows and columns or rows with no patient id
df <- df %>% janitor::remove_empty(., 'rows')
df <- df %>% janitor::remove_empty(., 'cols')

df <- df %>% filter(., !is.na(patient_number))

## arrange by patient number
df <- df %>% arrange(., patient_number)

df <- df %>% gather(., key, value, contains('day_'))

## extract day and type
df$day <- str_extract(df$key, '^day_.+_') %>% str_extract(., '\\d+') %>%
as.numeric()

df$key <- case_when(str_detect(df$key, 'steps_taken') ~ 'steps_taken',
                    str_detect(df$key, 'noted_ambulatory_status') ~
'noted_ambulatory_status',
                    str_detect(df$key, 'distance_traveled') ~
'distance_traveled',
                    str_detect(df$key, 'order_daily_ambulation') ~
'order_daily_ambulation'
)

## remove the rows with no values for value

df <- df %>% filter(., !is.na(value))


## •    For patient SID 60 - The discharge date is 2/15/2017 (I looked at the
excel sheet and it looks like admission date and surgery date were switched)
## •    For patient SID 56 - The surgery date for this patient: 1/19/2017,
discharge date: 1/21/2017 (Also looks like the dates were switched for this
patient)

## make the correction on the data per research coordinators' email

df$surgery_date[df$patient_number == 60] <- lubridate::mdy('02/13/2017')
df$discharge_date[df$patient_number == 60] <- lubridate::mdy('02/15/2017')

df$surgery_date[df$patient_number == 56] <- lubridate::mdy('1/19/2017')
df$discharge_date[df$patient_number == 56] <- lubridate::mdy('1/21/2017')

# -Day of admission is day one
# -In the excel day of admission is listed as Day 0 but it counts towards
length of stay
# -In the example you provided (i.e., 10/17 - 10/20) he said LOS is 4 days
```

© 2019 Daskivich TJ et al. *JAMA Network Open*.

```
df$length_of_stay <- as.numeric(df$discharge_date - df$admission_date) + 1

length(unique(df$patient_number))

## remove the rows with no discharge date
df <- df %>% filter(., !is.na(discharge_date))

length(unique(df$patient_number))

## excluded patient with greater than 30 days LOS
df <- df %>% filter(., length_of_stay < 30)

length(unique(df$patient_number))

## excluded the esophagectomy patient, only single patient in category
df <- df %>% filter(.,
type_of_surgery_0_esophagectomy_1_lung_lobectomy_2_gastric_bypass_3_hip_repla
cement_4_robotic_cystectomy_5_open_colectomy_6_abdominal_hysterectomy_7_sleev
e_gastrectomy_8_lap_colectomy != 0)

length(unique(df$patient_number))

## rename the surgery types labels to something more bearable
df <- df %>%
  rename(.,
         'surgery_type' =
type_of_surgery_0_esophagectomy_1_lung_lobectomy_2_gastric_bypass_3_hip_repla
cement_4_robotic_cystectomy_5_open_colectomy_6_abdominal_hysterectomy_7_sleev
e_gastrectomy_8_lap_colectomy,
         'race' =
race_ethnicity_0_caucasian_1_african_american_2_hispanic_3_asian_american_4_m
iddle_eastern)

## guess the data types
df <- modify(df, ~ parse_guess(.x))

df$day <- factor(df$day)

write_rds(df, 'data/processed_data.rds')

###########################################################
###########################################################
###########################################################

library(tidyverse)
library(magrittr)

theme_set(

  # cowplot::theme_cowplot() +
  theme_bw() +
    theme(panel.grid = element_blank())
)

df <- read_rds('data/processed_data.rds')

surgery_labels <- c('1' = 'Lung Lobectomy',
```

© 2019 Daskivich TJ et al. *JAMA Network Open*.

```
                    '2' = 'Gastric Bypass',
                    '3' = 'Hip Replacement',
                    '4' = 'Robotic Cystectomy',
                    '5' = 'Open Colectomy',
                    '6' = 'Abdominal Hysterectomy',
                    '7' = 'Sleeve Gastrectomy',
                    '8' = 'Laparoscopic Colectomy')

order_ambulation_labels <- c('0' = 'Bedrest',
                             '1' = 'OOB to Chair',
                             '2' = 'Ambulate with Assist',
                             '3' = 'Ambulate w/o Restriction')

noted_ambulation_label <- c('0' = 'No Ambulation',
                            '1' = 'OOB to Chair',
                            '2' = 'Ambulated QD',
                            '3' = 'Ambulated BID',
                            '4' = 'Ambulated TID',
                            '5' = 'Ambulated Ad lib')


## Figure 1
dat <- df %>% filter(., key == 'steps_taken')

fig1 <- ggplot(dat, aes(x = day, y = value)) +
  geom_boxplot(outlier.shape = '') +
  geom_jitter(width = .1, aes(color = factor(surgery_type))) +
  labs(x = 'Post Operative Day', y = 'Steps Taken') +
  scale_color_discrete(labels = surgery_labels) +
  theme(legend.position = 'bottom',
        legend.title = element_blank(),
        axis.title = element_text(size = 15, face = 'bold'))

fig1

ggsave('figures/fig1.eps', fig1, width = 7, height = 5)
ggsave('figures/fig1.jpg', fig1, width = 7, height = 5)

## Figure 2
dat <- df %>% filter(., key == 'steps_taken')

dat <- dat %>% filter(., surgery_type %in% c(4, 5, 8, 6))

fig2 <- ggplot(dat, aes(x = day, y = value)) +
  facet_wrap(~surgery_type, ncol = 2, labeller = labeller(surgery_type =
as_labeller(surgery_labels))) +
  geom_boxplot() +
  # coord_cartesian(ylim = c(0, 6000)) +
  labs(x = 'Post Operative Day', y = 'Steps Taken') +
  theme(axis.title = element_text(size = 15, face = 'bold'),
        axis.text = element_text(size = 10),
        strip.text = element_text(size = 10, face = 'bold'))

ggsave('figures/fig2.eps', fig2, width = 7, height = 5)
ggsave('figures/fig2.jpg', fig2, width = 7, height = 5)

## Figure 3
```

© 2019 Daskivich TJ et al. *JAMA Network Open*.

```
dat <- df %>% filter(., key %in% c('order_daily_ambulation', 'steps_taken'))
%>% spread(., key, value) %>% filter(., !is.na(order_daily_ambulation))

fig3a <- ggplot(dat, aes(x = factor(order_daily_ambulation), y =
steps_taken)) +
  geom_boxplot(outlier.shape = '') +
  scale_x_discrete(labels = order_ambulation_labels) +
  geom_jitter(width = .1, aes(color = factor(surgery_type))) +
  theme(axis.title = element_text(size = 15, face = 'bold'),
        axis.text = element_text(size = 9),
        strip.text = element_text(size = 10, face = 'bold'),
        legend.position = 'none',
        legend.title = element_blank()) +
  labs(x = 'Ordered Ambulation', y = 'Steps Taken') +
  scale_color_discrete(labels = surgery_labels)

dat <- df %>% filter(., key %in% c('noted_ambulatory_status', 'steps_taken'))
%>% spread(., key, value) %>% filter(., !is.na(noted_ambulatory_status))

fig3b <- ggplot(dat, aes(x = as.character(noted_ambulatory_status), y =
steps_taken)) +
  geom_boxplot(outlier.shape = '') +
  scale_x_discrete(labels = noted_ambulation_label) +
  geom_jitter(width = .1, aes(color = factor(surgery_type))) +
  theme(axis.title = element_text(size = 15, face = 'bold'),
        axis.text = element_text(size = 9),
        strip.text = element_text(size = 10, face = 'bold'),
        legend.position = 'bottom',
        legend.title = element_blank()) +
  labs(x = 'Physican Estimate of Ambulation', y = 'Steps Taken') +
  scale_color_discrete(labels = surgery_labels)

fig3 <- cowplot::plot_grid(fig3a, fig3b, ncol = 1, labels = 'AUTO',
label_size = 20)

cowplot::save_plot('figures/fig3.eps', fig3, base_height = 10, base_width =
7)
cowplot::save_plot('figures/fig3.jpg', fig3, base_height = 10, base_width =
7)

##


dat <- df %>% filter(., key %in% c('noted_ambulatory_status', 'steps_taken'))
%>% spread(., key, value) %>% filter(., !is.na(noted_ambulatory_status))


dat

ggplot(dat, aes(x = as.character(noted_ambulatory_status), y = steps_taken))
+
  geom_boxplot(outlier.shape = '') +
  scale_x_discrete(labels = noted_ambulation_label) +
  geom_jitter(width = .1, aes(color = factor(surgery_type))) +
  theme(axis.title = element_text(size = 15, face = 'bold'),
        axis.text = element_text(size = 10),
```

© 2019 Daskivich TJ et al. *JAMA Network Open*.

```
        strip.text = element_text(size = 10, face = 'bold'),
        legend.position = 'bottom',
        legend.title = element_blank()) +
  labs(x = 'Ambulatory Status', y = 'Steps Taken') +
  scale_color_discrete(labels = surgery_labels) +
  facet_wrap(~surgery_type)


## obtain sd estimates by surgery type and noted ambulation category
#
# dat %>%
#   group_by(., surgery_type, noted_ambulatory_status) %>%
#   nest() %>%
#   arrange(., surgery_type) %>%
#   mutate(., sd = map(data, ~ sd(.x$steps_taken))) %>%
#   select(., surgery_type, noted_ambulatory_status, sd) %>%
#   mutate(., surgery_type = factor(surgery_type, labels = surgery_labels))
%>%
#   mutate(., noted_ambulatory_status = factor(noted_ambulatory_status,
labels = noted_ambulation_label)) %>%
#   unnest() %>% write_csv(.,
'sd_errors_by_surgery_type_and_noted_ambulation.csv', na = '')

#####################################################################
#####################################################################
#####################################################################

library(tidyverse)
library(rms)

## set global theme
theme_set(
  theme_bw()
)

## read the data
df <- read_rds('data/processed_data.rds')

# labels of surgeries
surgery_labels <- c('1' = 'Lung Lobectomy',
                    '2' = 'Gastric Bypass',
                    '3' = 'Hip Replacement',
                    '4' = 'Robotic Cystectomy',
                    '5' = 'Open Colectomy',
                    '6' = 'Abdominal Hysterectomy',
                    '7' = 'Sleeve Gastrectomy',
                    '8' = 'Laprascopic Colectomy')

## filter data for steps taken data
dat <- df %>% filter(., key == 'steps_taken')

## make into factor
dat$surgery_type <- factor(dat$surgery_type, labels = surgery_labels)

## determine cutoff for 70th, 80th, and 90th quantile on per surgery basis
results_summary_stats <- dat %>%
  group_by(., surgery_type, day) %>%
```

© 2019 Daskivich TJ et al. *JAMA Network Open*.

```
  nest() %>%
  arrange(., surgery_type) %>%
  mutate(.,
         q70 = map_dbl(data, ~ quantile(.x$length_of_stay, .70)),
         q80 = map_dbl(data, ~ quantile(.x$length_of_stay, .80)),
         q90 = map_dbl(data, ~ quantile(.x$length_of_stay, .90)),
  ) %>%
  select(., surgery_type, day, q70, q80, q90) %>%
  unnest()

## merge cutoff data back with main dataset
dat <- dat %>% left_join(., results_summary_stats)

## identify patients with los exceeding cutoff
dat$long_los_70 <- factor(ifelse(dat$length_of_stay >= dat$q70, 1, 0))
dat$long_los_80 <- factor(ifelse(dat$length_of_stay >= dat$q80, 1, 0))
dat$long_los_90 <- factor(ifelse(dat$length_of_stay >= dat$q90, 1, 0))

## subset the data to only certain variables
dat <- dat %>% select(., patient_number, race, surgery_type,
charlson_comorbidity_index_score, bmi, age, sex_0_male_1_female,
contains('long_'), day, value)

## only subset for day 1 data and day 2 data
dat1 <- dat %>% filter(., day == 1)
dat2 <- dat %>% filter(., day == 2)


## make models
dd <- datadist(dat1)
options(datadist = 'dd')

## model estimates of full model
summary(fit <- glm(long_los_70 ~ rcs(value, 3) + age + sex_0_male_1_female +
race + charlson_comorbidity_index_score + surgery_type, data = dat1, family =
'binomial'))

## model estimates of stepwise reduced model
summary(MASS::stepAIC(fit))

## plot model estimates
fit <- lrm(long_los_70 ~ rcs(value, 3) + surgery_type, data = dat1, x = T, y
= T)
fig4a <- ggplot(datestimate <- Predict(fit, value, fun = function(x) exp(x) /
(1+exp(x))))
fig4a <- fig4a + coord_cartesian(ylim = c(0, 1), xlim = c(0, 2500)) +
  labs(x = 'Steps Taken', y = 'Predicted Probabilities', caption = '', title
= 'Day 1 - 70th Percentile Cutoff') +
  scale_x_continuous(breaks = seq(0, 2500, 500)) +
  scale_y_continuous(labels = scales::percent) +
  theme(legend.position = 'none',
        legend.title = element_blank(),
        axis.title = element_text(size = 10, face = 'bold'),
        axis.text = element_text(size = 10),
        panel.grid = element_blank())
```

© 2019 Daskivich TJ et al. *JAMA Network Open*.

```
fit <- lrm(long_los_70 ~ rcs(value, 3) + surgery_type, data = dat1, x = T, y
= T)
fig4b <- ggplot(datestimate <- Predict(fit, value, surgery_type, fun =
function(x) exp(x) / (1+exp(x)), conf.int = F))
fig4b <- fig4b + coord_cartesian(ylim = c(0, 1), xlim = c(0, 2500)) +
  labs(x = 'Steps Taken', y = 'Predicted Probabilities', caption = '', title
= 'Day 1 - 70th Percentile Cutoff') +
  scale_x_continuous(breaks = seq(0, 2500, 500)) +
  scale_y_continuous(labels = scales::percent) +
  theme(legend.position = 'none',
        legend.title = element_blank(),
        axis.title = element_text(size = 10, face = 'bold'),
        axis.text = element_text(size = 10),
        panel.grid = element_blank())


fit <- lrm(long_los_80 ~ rcs(value, 3) + surgery_type, data = dat1, x = T, y
= T)
fig4c <- ggplot(datestimate <- Predict(fit, value, fun = function(x) exp(x) /
(1+exp(x))))
fig4c <- fig4c + coord_cartesian(ylim = c(0, 1), xlim = c(0, 2500)) +
  labs(x = 'Steps Taken', y = 'Predicted Probabilities', caption = '', title
= 'Day 1 - 80th Percentile Cutoff') +
  scale_x_continuous(breaks = seq(0, 2500, 500)) +
  scale_y_continuous(labels = scales::percent) +
  theme(legend.position = 'none',
        legend.title = element_blank(),
        axis.title = element_text(size = 10, face = 'bold'),
        axis.text = element_text(size = 10),
        panel.grid = element_blank())


fit <- lrm(long_los_80 ~ rcs(value, 3) + surgery_type, data = dat1, x = T, y
= T)
fig4d <- ggplot(datestimate <- Predict(fit, value, surgery_type, fun =
function(x) exp(x) / (1+exp(x)), conf.int = F))
fig4d <- fig4d + coord_cartesian(ylim = c(0, 1), xlim = c(0, 2500)) +
  labs(x = 'Steps Taken', y = 'Predicted Probabilities', caption = '', title
= 'Day 1 - 80th Percentile Cutoff') +
  scale_x_continuous(breaks = seq(0, 2500, 500)) +
  scale_y_continuous(labels = scales::percent) +
  theme(legend.position = 'bottom',
        legend.title = element_blank(),
        axis.title = element_text(size = 10, face = 'bold'),
        axis.text = element_text(size = 10),
        panel.grid = element_blank())

fig4d

ggsave('legend.tiff',fig4d,  dpi = 300)
fig4 <- cowplot::plot_grid(fig4a, fig4c, fig4b, fig4d, labels = 'AUTO')

ggsave('figures/fig4.jpg', fig4, height = 8, width = 10)
ggsave('figures/fig4.tiff', fig4, height = 8, width = 10, dpi = 300)

## determine effects less than 1k steps and > 1k steps
```

© 2019 Daskivich TJ et al. *JAMA Network Open*.

```
fit <- lrm(long_los_70 ~ I(value/100) + surgery_type, data = dat1, subset =
value < 1000)
fit
summary(lrm(long_los_70 ~ I(value/100) + surgery_type, data = dat1, subset =
value < 1000), value = c(0, 100))

fit <- lrm(long_los_70 ~ I(value/100) + surgery_type, data = dat1, subset =
value > 1000)
fit
summary(lrm(long_los_70 ~ I(value/100) + surgery_type, data = dat1, subset =
value > 1000), value = c(0, 100))


###########################################################
###########################################################
###########################################################

pearson_correlation_results <- df %>%
  group_by(., surgery_type) %>%
  filter(., key == 'steps_taken') %>%
  nest() %>%
  arrange(., surgery_type) %>%
  mutate(., cor_test = map(data, ~ cor.test(as.numeric(as.character(.x$day)),
.x$value))) %>%
  mutate(., results = map(cor_test, ~ broom::tidy(.x))) %>%
  select(., surgery_type, results) %>%
  unnest()

pearson_correlation_results %>% View()

p_bartlett <- possibly(bartlett.test, NA)

bartlett_test_results <- df %>%
  group_by(., surgery_type) %>%
  filter(., key == 'steps_taken') %>%
  nest() %>%
  arrange(., surgery_type) %>%
  mutate(., results = map(data, ~ bartlett.test(.x$value, factor(.x$day))))
%>%
  mutate(., results = map(results, ~ broom::tidy(.x))) %>%
  select(., surgery_type, results) %>%
  unnest()
```

© 2019 Daskivich TJ et al. *JAMA Network Open*.
